# Negative multiparametric magnetic resonance imaging for prostate cancer: further outcome and consequences

**DOI:** 10.1007/s00345-022-04197-8

**Published:** 2022-11-01

**Authors:** Maximilian Haack, Vanessa Miksch, Zhe Tian, Gregor Duwe, Anita Thomas, Angelika Borkowetz, Kristina Stroh, Christian Thomas, Axel Haferkamp, Thomas Höfner, Katharina Boehm

**Affiliations:** 1 Department of Urology and Pediatric Urology, Johannes-Gutenberg University Medical Center, Mainz, Germany; 2 Department of Urology and Pediatric Urology, Carl-Gustav-Carus University Medical Center, Dresden, Germany; 3 Department of Epidemiology, Biostatistics and Occupational Health, McGill University, Montreal, Canada; 4 Department of Diagnostic and Interventional Radiology, Johannes-Gutenberg University Medical Center, Mainz, Germany

**Keywords:** Prostate (carcinoma), Prostate biopsy, MRI of the prostate, PI-RADS

## Abstract

**Purpose:**

EAU guidelines recommend multiparametric MRI of the prostate (mpMRI) prior to biopsy to increase accuracy and reduce biopsies. Whether biopsy can be avoided in case of negative mpMRI remains unclear. Aim of this study is to evaluate predictors of overall prostate cancer (PCa) in negative mpMRI.

**Methods:**

A total of 216 patients from 2018 to 2020 with suspicion of PCa and negative mpMRI (PI-RADS ≤ 2) were interviewed by telephone about outcome and further follow-up. Clinically significant PCa (csPCa) was defined as ISUP ≥ 2. Patients with vs. without biopsy and with vs. without PCa were compared. Univariate and multivariate analyses were performed to evaluate predictors of PCa occurrence in patients with negative mpMRI.

**Results:**

15.7% and 5.1% of patients with PI-RADS ≤ 2 on mpMRI showed PCa and csPCa, respectively. PCa patients had higher PSAD (0.14 vs. 0.09 ng/ml^2^; *p* = 0.001) and lower prostate volume (50.5 vs. 74.0 ml; *p* = 0.003). Patients without biopsy (25%) after MRI were older (69 vs. 65.5 years; *p* = 0.027), showed lower PSA (5.7 vs. 6.73 ng/ml; *p* = 0.033) and lower PSA density (0.09 vs. 0.1 ng/ml^2^; *p* = 0.027). Multivariate analysis revealed age (OR 1.09 [1.02–1.16]; *p* = 0.011), prostate volume (OR 0.982 [0.065; 0.997]; *p* = 0.027), total PSA level (OR 1.22 [1.01–1.47], *p* = 0.033), free PSA (OR 0.431 [0.177; 0.927]; *p* = 0.049) and no PI-RADS lesion vs PI-RADS 1–2 lesion (OR 0.38 [0.15–0.91], *p* = 0.032.) as predictive factors for the endpoint presence of PCa.

**Conclusions:**

Biopsy for selected patient groups (higher age, prostate volume and free PSA as well as lower PSA-Density) with negative mpMRI can be avoided, if sufficient follow-up care is guaranteed. Detailed counseling regarding residual risk for undetected prostate cancer should be mandatory.

## Introduction

Since the introduction and widespread use of MRI targeted prostate biopsies, detection rates increased over the past years [1]. Thus, EAU-guidelines on prostate cancer recommend to perform multiparametric MRI (mpMRI) prior to prostate biopsy [2]. But there remains great dispute over biopsy necessity in MRI-negative patients with clinical suspicion for PCa. On one hand, patients and clinicians strongly desire diagnostic clarification which mostly leads to a systematic prostate biopsy when MRI results show negative. On the other hand, there is fear on both sides of overevaluation and overdiagnosis which leads to unnecessary biopsies or overtreatment of non-clinically significant PCa (ISUP = 1). Therefore, there is a strong demand for additional prognostic factors in MRI-negative patients with suspicion of PCa to take a more differentiated decision toward prostate biopsy. Alongside total PSA levels, PSA density, free/total PSA ratio and PSA doubling time are additional parameters typically used for PCa management and surveillance. But there are no sufficient data that recommend either of those factors for diagnostic use prior to prostate biopsy [3–6], due to multiple variations (such as prostate volume, different intervals between PSA determination, variables in PSA measurement, instability of PSA at different temperatures). In addition, these factors were not evaluated in patients with negative MRI. Evaluation of blood- or urine-based biomarkers is still in an experimental phase and not widely accessible. Therefore, further investigation of patients with negative mpMRI and clinical suspicion for PCa is needed. In this study, patients’ outcomes after initial negative mpMRI were collected over 3 years, to investigate undetected clinically significant PCa (csPCa) and subsequent therapy, such as prostate cancer treatment, deobstruction therapy and further diagnostic evaluation via MRI.

## Patients and methods

### Study design and population

1380 prostate mpMRI were performed at our center between January 2018 and December 2020. For this retrospective observational study, 255 men showing a PI-RADS-classification ≤ 2 were reviewed regarding evolution after negative mpMRI with a median follow-up of 21.5 months. After approval of the local ethics committee, patients not being followed at our center were contacted and supplementary data (PSA levels, further mpMRI, biopsies, prostate surgery, diagnosis of PCa and following therapy) were collected via phone interview. 39 patients were excluded due to diagnosis of PCa before inclusion MRI (19 patients), experimental treatment of BPH or PCa (12), refusal of participation (5) and residence abroad (3). Finally, 216 patients were analyzed.

### mpMRI

From all 1380 mpMRI, 1372 were realized using a 3-Tesla MRI, 8 using a 1.5-Tesla MRI. The MRI inquisition protocol included T1-weighted imaging (T1WI), T2-weighted imaging (T2WI), diffusion weighted imaging (DWI) and Dynamic contrast-enhanced MR imaging (DCE-MRI). mpMRI studies at our center were assessed using the Prostate Imaging and Reporting Data System (PI-RADS) version 2 and 2.1, respectively. According to both versions, MRI was considered negative with a classification ≤ 2. Prostate volume, limitation **in** assessability, number of lesions (0–4), number of index lesions (PI-RADS ≥ 3) in follow-up MRI, PI-RADS classification system and localization for each lesion and auxiliary findings were registered. As we included external mpMRI to depict a realistic clinical setting, no consistent MRI protocol was applied to former and further mpMRI taken outside of our center. Nevertheless, all mpMRI were evaluated using the PI-RADS classification.

### Prostate biopsy and clinically significant cancer

In case of PI-RADS classification ≤ 2, a systematic 12-fold biopsy was performed at our center as transrectal ultrasound (TRUS) guided biopsy under local anesthesia and perioperative antibiotic prophylaxis (Table 1), with 6 biopsies from each side and two (1 × medially and 1 × laterally) in three levels (basal, mid and apical). 24-fold biopsies were performed, if the first 12-fold biopsy was negative and following mpMRI was also negative. These saturation-biopsies were also performed systematically. If following mpMRI showed suspicious lesions, an MRI/TRUS fusion biopsy was performed using *Hi ViSiOn Ascendus* by Hitachi Medical Systems. Hereby, the targeted biopsies (2–4 per target) were taken from the suspicious lesion and a systematic 12-fold biopsy was performed as well. Biopsies taken outside our center were more heterogeneous but were included to represent a more realistic clinical scenario and increase inclusion rate with patients not necessarily followed-up at a university hospital for all diagnostics and follow-up. csPCa was defined as ISUP ≥ 2 based on biopsy histology.

### Statistical analysis

Univariate and multivariate regression analyses were performed to identify independent predictors for PCa despite negative mpMRI (Table 3). All data were analyzed using RStudio (Version 2021.09.0) software. Statistical significance was defined as *p* < 0.05.

## Results

### Study population

In Table 1, the patients’ characteristics and outcome are shown. Mean patient age at mpMRI was 66 years (Interquartile range (IQR)) 58; 71), mean PSA level prior to mpMRI was 6.5 ng/ml (IQR 4.97; 9.20), free PSA level 1.36 ng/ml (IQR 0.88; 2.12), PSA density 0.1 ng/ml^2^ (IQR 0.07; 0.14) and prostate volume 68.5 ml (IQR 47.7; 100). Sixty-three (29.2%) men had received negative biopsy prior to initial mpMRI (Table 1).

### Biopsy after negative mpMRI

In total, 162 (75%) patients underwent biopsy, from which 133 (82.1%) received systematic 12-fold biopsy, 14 (8.64%) systematic 24-fold biopsy and two (1.23%) targeted biopsy after suspicion in a further mpMRI (Table 2).

Univariate analysis showed that patients not undergoing biopsy were significantly older when undergoing initial mpMRI (69.0 versus 65.5 years; Odds Ratio (OR) 1.04, 95%-confidence interval (CI) [1.00; 1.08], *p* = 0.027), had lower initial PSA level (5.7 versus 6.73 ng/ml; OR 0.94, CI [0.86; 1.01], *p* = 0.033) and lower PSA density (0.09 versus 0.1 ng/ml^2^; OR 0.01, CI [0.00; 1.43], *p* = 0.027) (Table 2). Patients not undergoing biopsy received treatment for BPH significantly more often than those undergoing biopsy (25.9% versus 21%, *p* = 0.007) (Table 2).

In multivariate analysis, age at initial mpMRI showed to be an independent predictive factor for performing biopsy after mpMRI (OR 0.907, CI [0.853; 0.959], *p* = 0.001], whereas PSA, free PSA and PSA density did not (Table 2). Neither a negative biopsy prior to mpMRI nor absence of PI-RADS lesions in mpMRI turned out to be independent predictive factors of performing biopsy after negative mpMRI (Table 2).

### PCa/csPCa despite negative mpMRI

After a mean follow-up of 21.5 (IQR 14.5; 28.0) months, we found that 20.7% (34/164) of patients who received biopsy showed prostate carcinoma and only 6,7% (11/164) showed clinically significant prostate cancer, from which just one was high risk (Gleason 9). However, we had a closer look at patients without previous biopsy and found that 3.7% (2/54) patients were diagnosed with prostate cancer. Both patients harbored significant PCa (Gleason 7 and Gleason 9). In patients with previous biopsy, 5.5% (9/162) patients were diagnosed with significant PCa and 19.5% (32/164) were diagnosed with any PCa. Risk factors for positive biopsy were similar to the entire cohort. Eighteen patients (8.3% of the study cohort, 52.9% of patients being diagnosed with PCa) received active therapy, from which 15 (6.9%/44.1%) radical prostatectomy (RPx), subsequently (Table 3).

Performing univariate analysis, PSA density showed to be a predictive factor for PCa in patients with negative mpMRI (0.14 versus 0.09 ng/ml/cm^3^; OR 1624, CI [15.9; 165689], *p* = 0.001), whereas total PSA value itself did not (OR 0.99, CI [0.92; 1.06], *p* = 0.569) (Table 3). Furthermore, patients who showed PCa subsequently had lower prostate volume (50.5 versus 74.0 ml; OR 0.99, CI [0.98; 1.00], *p* = 0.003) (Table 3). Interestingly, patients with PCa had shown signs of prostatitis in initial mpMRI significantly more often than those without PCa (14 (41.2%) versus 32 (17.6%); OR 3.26, CI [1.47; 7.17], *p* = 0.004) (Table 3).

Multivariate analysis revealed that age at initial mpMRI (OR 1.09 [1.02–1.16]; *p* = 0.011), prostate volume (OR 0.982 [0.065; 0.997]; *p* = 0.027), total PSA level (OR 1.22 [1.01–1.47], *p* = 0.033) and free PSA (OR 0.431 [0.177; 0.927]; *p* = 0.049) were independent predictive factors for the occurrence of PCa (Table 3). Furthermore, it showed that patients who had not shown PI-RADS lesions at all were less likely to have or develop PCa compared to those who had shown unsignificant PI-RADS lesions (classified with 1 or 2) in initial mpMRI (OR 0.38 [0.15–0.91], *p* = 0.032.) (Table 3). Men without PI-RADS lesions had significantly lower initial PSA levels (5.9 versus 6.69 ng/ml; OR 0.96, CI [0.91; 1.01], *p* = 0.044), and follow-up was shorter (17.7 versus 25.2 months (OR 0.92, CI [0.90; 0.96], p ≤ 0.001) (Data not shown).

## Discussion

With prostate cancer being the most common malign tumor disease in men, there is great demand for more diagnostic certainty in patients with negative mpMRI to avoid over diagnostic. At our medical center, 15.65% (216 eligible patients out of 1380 overall) between 2018 and 2020 showed negative mpMRI, from which 75% received prostate biopsy with only 5.1% of them showing clinically significant prostate cancer. Previous studies showed negative predictive values (NPV) of mpMRI, ranging from 76 to 99% [7–23]. Thus, the negative prognostic value of mpMRI at our institute is sufficient and congruent to other data. But the remaining uncertainty on both sides (patient and clinician) leads to anxiety and subsequently to overevaluation and unnecessary biopsies. Clinical and laboratory factors such as family history, digital rectal examination, total PSA, PSA density, PSA velocity, Biomarkers (e.g., PCA3, PHI, 4Kscore) and BRCA mutation analysis can support the decision making progress regarding prostate cancer evaluation with prostate biopsy and help in the argumentation against overtreatment. Unfortunately, most of these factors are still being investigated and partly show incongruent data (in case of total PSA, age or prostate volume) [8, 10, 15, 21, 24, 25].

Due to the wide accessibility, PSA density is one of the most investigated predictive factors in addition to mpMRI and can elevate the NPV of mpMRI significantly [17, 24]. Schoots et al. developed a guidance tool for biopsy decision making in biopsy-naïve patients dependent on PSA Density [26]. They recommend not performing biopsy if PSA Density is < 0.20 ng/ml/cm^3^ and PI-RADS Score is 1 or 2 in low and intermediate risk category [26]. Our data show a significant positive correlation (OR 1624, CI [15.9; 165689]), *p* = 0.001) in patients with higher PSA density for the occurrence of PCa (Table 3). Interestingly, prostate volume and total PSA level prior to mpMRI showed to be independent predictive factors for PCa (Table 3). Therefore, patients with smaller prostate volume and higher PSA showed PCa significantly more often. As PSA density is calculated from total PSA in relation to prostate volume, it was surprising to see that PSA density was not an independent predictive factor. Interestingly total PSA was slightly higher (6.55 ng/ml) in non-PCa patients (in comparison with 6.4 ng/ml) (Table 3). This effect might be caused by patient selection, as only patients with elevated PSA-level received MRI evaluation. Free PSA also showed to be an additional independent predictive factor (Table 3). Free PSA usually is related with bound PSA to create the f/t-PSA-quotient. The lower the free PSA, the higher the probability of PCa. In this study, free PSA level was significantly lower (1.08 ng/ml) in patients with PCa than in non-PCa (1.47 ng/ml) and therefore, congruent to this relation.

Age is a commonly known risk factor for PCa. Consistently to that age showed to be an independent predictive factor for PCa as well (67 versus 66 years) (Table 3). Considering decision making for prostate biopsy, only age could be identified as an independent predictive factor (Table 2). Total PSA prior to mpMRI and PSA density showed significant predicational value in univariate analysis, which, however, could not be reproduced in multivariate analysis (Table 2). Investigation of secondary findings described in the initial mpMRI showed that patients who were subsequently diagnosed with PCa showed signs of prostatitis significantly more often than men without (41.2 versus 17.6%; OR 3.26, CI [1.47; 7.17], *p* = 0.004) (Table 3). Other secondary findings considered (BPH, post-infectious findings and calcifications) did not turn out to be statistically significant risk factors. It must be noted that only the presence or absence of an incidental finding was recorded in the initial mpMRI as part of the data collection. Whether the secondary findings corresponded in their localization to a subsequently diagnosed prostate carcinoma was not further examined. Other studies found prostatitis to be a risk factor for undetected prostate cancer [24, 27, 28]. However, the current data are not sufficient to make a general recommendation for a systematic biopsy in the case of a negative mpMRI with signs of prostatitis.

These data may reflect the existing uncertainty of treating physicians to decide against an unnecessary biopsy. However, considering the data from Table 3, the decision against a biopsy could be made easier due to the independent predictive power of age, PSA, free PSA, prostate volume and PI-RADS lesion in relation to the occurrence of prostate cancer.

In summary, our study provides significant data that support the predictive value of the mpMRI as well as clinical and PSA-related factors, specifically in patients with negative mpMRI. With further investigation, these parameters could become mandatory in the decision process regarding prostate biopsies in cases with negative mpMRI to elevate the negative predictive value of mpMRI even more. In selected patient groups (older patients, higher prostate volume, higher free PSA and lower PSA-Density), prostate biopsy after negative mpMRI can be avoided due to lower risk of undetected prostate cancer. But counseling of these patients regarding residual risk for undetected prostate cancer and an offer for a prostate biopsy should be mandatory. Biomarkers could provide more diagnostic certainty. However, many candidates still require extensive research and are bound to be less accessible in the near future, because PSA and mpMRI are current fundamental diagnostics for PCa. Additional imaging procedures as high frequent ultrasound may help to reduce these problems even more.

Our study also has some limitations. Data acquisition was retrospective, and study population was relatively small. Also, MRI machines and PI-RADS Versions varied in the observation period. Furthermore, prostate biopsies differed in biopsy counts. There is no fixed definition of csPCa, which limits the comparability to other studies.

In addition, it is unclear how many undetected PCa could have been missed in the none biopsy group (52 out of 216). This reflects the real-life scenario in which patients and clinicians find themselves in. This group of patients who never received a biopsy either rejected biopsy or showed stable follow-up (constant PSA, no PI-RADS lesions in following mpMRI, etc.). It is also arguable if a 12-fold systematic biopsy is sufficient as “gold standard” as a significant proportion of PCa is missed by this diagnostic procedure. In our opinion, our endpoint “no diagnosis of PCa” is reasonable, since the risk of undetected clinically significant prostate carcinoma in this specific group of patients is low. Therefore, we think it is justifiable not to perform prostate biopsy, if sufficient follow-up care is guaranteed. A major concern remains that the follow-up time of 21 months might not be long enough to fully answer this question without performing biopsy in all patients.

Further information on the entire patient cohort, including men with PIRADS 3–5, would be of value. However, the goal of this specific study was to depict real-time scenarios where some patients had unclear MRI results. Specifically, we aimed to dig deeper in those patients with no PIRADS lesion or PIRADS 1 and 2 lesions. Therefore, we conducted telephone interviews within our patient cohort. Due to economic and staff related shortcomings, it was not possible to perform this detailed work up for all patients. Therefore, we do not have data of the 1120 mpMRI which showed suspicious lesions (PI-RADS ≥ 3). Therefore, we cannot investigate, on how many of these patients subsequently had prostate cancer.

We think that our follow-up is comparatively consistent due to telephone interviews on all patients who were not followed-up in our clinic. We understand that our study suffers from lack of standardization, randomization, controls and selection bias, due to the retrospective analysis of real-world data.

## Conclusion

The additional evaluation of clinical factors such as total and free PSA, age and prostate volume in combination with mpMRI could increase the diagnostic certainty in patients with negative mpMRI with regard to non-significant prostate cancers in order to reduce unnecessary prostate biopsy. Patients with negative MRI are in sum at low risk of csPCa.

## Appendix

See Tables 1, 2 and 3.

**Table 1:** Patients’ characteristics and outcome

Patients	*n* = 216 mean [IQR]
Age at MRI [years]	66.0 [58.0;71.0]
PSA level prior to MRI [ng/ml]	6.50 [4.97;9.20]
Free PSA prior to MRI [ng/ml]	1.36 [0.88;2.12]
PSA density [ng/ml^2^]	0.10 [0.07;0.14]
Prostate volume [ml]	68.5 [47.7;100]
	*n* (%)
Bx prior to MRI	63 (29.2)
Bx after MRI	162 (75)
Systematic 12-fold	133
Systematic 24-fold	14
Targeted	2
Unclear	13
No Bx after MRI	54 (25)
Follow-up MRI	
0	194
1	20
2	1
3	1
PCa	34 (15.7)
csPCa	11 (5.1)

**Table 2:** Biopsy after negative mpMRI

	Biopsy *n* = 162 *n* (%)	No biopsy *n* = 54 *n* (%)	Univariate analysis			Multivariate analysis	
			OR [95% CI]	*p* ratio	*p* overall	OR [95% CI]	*p* value
Biopsy							
None	0 (0.00)	54 (100)					
Systematic 12-fold	133 (82.1)	0 (0.00)					
Systematic 24-fold	14 (8.64)	0 (0.00)					
Targeted	2 (1.23)	0 (0.00)					
Unclear	13 (8.02)	0 (0.00)					
Biopsy prior to inclusion MRI					0.545		
0	117 (72.2)	36 (66.7)	Ref				
1	45 (27.8)	18 (33.3)	1.30 [0.66;2.51]	0.441		0.782 [0.339;1.897]	0.58
PI-RADS lesion (1–2) in mpMRI					1.000		
0	87 (53.7)	29 (53.7)	1.00 [0.54;1.87]	0.998		0.845 [0.382;1.843]	0.674
1	75 (46.3)	25 (46.3)	Ref	Ref			
BPH therapy					0.007		
0	102 (63.0)	40 (74.1)					
1	34 (21.0)	14 (25.9)					
NA	26 (16.0)	0 (0.00)					
	**mean [IQR]**	**mean [IQR]**					
Age at MRI [years]	65.5 [58.0;70.0]	69.0 [61.2;74.0]	1.04 [1.00;1.08]	0.044	0.027	0.907 [0.853;0.959]	0.001
PSA prior to MRI [ng/ml]	6.73 [5.08;9.40]	5.70 [4.60;8.28]	0.94 [0.86;1.01]	0.098	0.033	1.088 [0.859;1.391]	0.488
Prostate Volume [ml]	68.5 [48.6;100]	70.7 [47.0;102]	1.00 [0.99;1.00]	0.490	0.861	1.013 [0.998;1.032]	0.138
Free PSA [ng/ml]	1.31 [0.90;2.06]	1.65 [0.79;2.19]	1.20 [0.90;1.59]	0.212	0.456	0.721 [0.430;1.137]	0.169
PSA Density [ng/ml^2^]	0.10 [0.07;0.15]	0.08 [0.06;0.11]	0.01 [0.00;1.43]	0.068	0.027	14.288 [0.000;10.314]	0.668

**Table 3:** PCa after negative mpMRI

	No PCa *n* = 182 *n* (%)	PCa *n* = 34 *n* (%)	Univariate analysis			Multivariate analysis	
			OR [95% CI]	*p* ratio	*p* overall	OR [95% CI]	*p* value
Biopsy							
None	52 (28.6)	2 (5.88)					
Systematic 12-fold	104 (57.1)	29 (85.3)					
Systematic 24-fold	11 (6.04)	3 (8.82)					
Targeted	2 (1.10)	0 (0.00)					
Unclear	13 (7.14)	0 (0.00)					
Biopsy prior to mpMRI					0.560		
0	127 (69.8)	26 (76.5)	Ref				
1	55 (30.2)	8 (23.5)	0.72 [0.29;1.64]				
Gleason score							
6		23 (67.6)					
7		10 (29.4)					
9		1 (2.94)					
NA	182 (100)						
D’Amico classification							
Intermediate risk		8 (23.5)					
Low risk		18 (52.9)					
No biopsy	52 (28.6)	2 (5.88)					
NA	130 (71.4)	6 (17.6)					
Active therapy							
0	182 (100)	16 (47.1)					
1		18 (52.9)					
RPx							
0	157 (86.3)	17 (50.0)					
1	0 (0.00)	15 (44.1)					
NA	25 (13.7)	2 (5.88)					
PI-RADS lesion (1–2) in mpMRI					0.075		
0	103 (56.6)	13 (38.2)	0.48 [0.22;1.01]	0.053		0.382 [0.153;0.908]	0.032
1	79 (43.4)	21 (61.8)	Ref				
Limited assessability of mpMRI					0.244		
0	163 (89.6)	28 (82.4)	Ref				
1	19 (10.4)	6 (17.6)	1.86 [0.62;4.89]	0.251			
Auxiliary findings in mpMRI: BPH					0.775		
0	21 (11.5)	3 (8.82)	Ref				
1	161 (88.5)	31 (91.2)	1.29 [0.41;5.97]	0.688			
Auxiliary findings in mpMRI: prostatitis					0.004		
0	150 (82.4)	20 (58.8)	Ref				
1	32 (17.6)	14 (41.2)	3.26 [1.47;7.17]	0.004			
Auxiliary findings in mpMRI: postinflammatory					0.594		
0	157 (86.3)	28 (82.4)	Ref				
1	25 (13.7)	6 (17.6)	1.37 [0.47;3.48]	0.547			
Auxiliary findings in mpMRI: calcification					1.000		
0	171 (94.0)	32 (94.1)	Ref				
1	11 (6.04)	2 (5.88)	1.03 [0.14;4.15]	0.973			
	**Mean [IQR]**	**Mean [IQR]**					
Age at mpMRI [years]	66.0 [58.0;71.0]	67.0 [59.2;73.8]	1.03 [0.98;1.08]	0.229	0.285	1.085 [1.021;1.160]	0.011
Prostate volume [ml]	74.0 [49.9;103]	50.5 [36.3;62.0]	0.99 [0.98;1.00]	0.028	0.003	0.982 [0.065;0.997]	0.027
PSA prior to mpMRI [ng/ml]	6.55 [4.90;9.20]	6.40 [5.43;9.02]	0.99 [0.92;1.06]	0.812	0.569	1.222 [1.013;1.471]	0.033
Free PSA [ng/ml]	1.47[0.92;2.18]	1.08 [0.80;1.70]	0.60 [0.36;1.00]	0.050	0.054	0.431 [0.177;0.927]	0.049
PSA density [ng/ml^2^]	0.09 [0.07;0.13]	0.14 [0.09;0.20]	1624 [15.9;165689]	0.002	0.001		
